# Cigarette ignition propensity, smoking behavior, and toxicant exposure: A natural experiment in Canada

**DOI:** 10.1186/1617-9625-9-13

**Published:** 2011-12-21

**Authors:** Kristie M June, David Hammond, Andreas Sjödin, Zheng Li, Lovisa Romanoff, Richard J O'Connor

**Affiliations:** 1 Department of Health Behavior, Roswell Park Cancer Institute, Elm and Carlton Streets, Buffalo, NY, 14263, USA; 2 Department of Health Studies and Gerontology, University of Waterloo, 200 University Avenue West, Waterloo, ON N2L 3G1 Canada; 3 National Center for Environmental Health, Centers for Disease Control and Prevention, 1600 Clifton Rd., Atlanta, GA 30333, USA

**Keywords:** Reduced Ignition Propensity (RIP) Cigarettes, Smoking Topography, Polycyclic Aromatic Hydrocarbons (PAH)

## Abstract

**Background:**

This study used a 'pre-post' research design to measure the impact of the Canadian reduced ignition propensity law on cigarette toxicity and smoking behavior among Canadian smokers.

**Method:**

The study was conducted in Ontario, Canada over a ten-month period in 2005-2006, consisting of 4 laboratory visits (baseline N = 61, final N = 42). At Visit 1, questionnaire data and biospecimens were collected. During the following 24 hours, participants smoked 5 cigarettes *ad libitum *through a topography recording device and collected their cigarette butts. Visit 2 consisted of a questionnaire and smoking one cigarette to measure laboratory topography values. After ten months, these procedures were repeated.

**Results:**

Generalized estimating equations, with law status (pre and post) as a fixed within-subject factor, were used to determine changes in behavior and biomarker exposure. Overall, there were no significant differences in smoking topography, breath carbon monoxide, and saliva cotinine pre-post law (*p*>0.1). However, analyses revealed a significant increase in the summed concentrations of hydroxyfluorene metabolites (N = 3),, and 1-hydroxypyrene in urine, with at notable increase in hydroxyphenanthrene metabolites (N = 3) (*p*_Σhydroxyfluorene _= 0.013, 22% increase; *p_1-hydroxypyrene _*= 0.018, 24% increase; *p*_Σhydroxyphenanthrene _= 0.061, 17% increase).

**Conclusion:**

While the results suggest no change in topography variables, data showed increases in exposure to three PAH biomarkers following reduced ignition propensity implementation in Canada. These findings suggest that human studies should be considered to evaluate policy impacts.

## Background

Cigarette fires are the leading cause of fire related deaths in Canada [1]. In 2002 alone, 9,414 fires were ignited by smokers' materials and open flames; resulting in 688 injuries and 94 deaths [2]. In 2005, Canada became the first country to legislate RIP (Reduced Ignition Propensity) cigarettes and the second jurisdiction following New York State's lead in mandating fire safety standards for all cigarettes sold in-state [3]. The Canadian law requires that cigarettes self-extinguish 75% of the time when tested under standard protocol and has been shown to reduce the ignition propensity of cigarettes [4-6]. Although Canadian law sets the standard on the ignition propensity of cigarettes, it does not stipulate how RIP cigarettes are achieved. While the technology to produce RIP cigarettes has been in place within the cigarette industry for decades [7], currently the most common method of compliance is the use of lower porosity bands applied to the cigarette paper [8]. These bands can best be described as 'speed bumps' that ultimately aid in extinguishing the cigarette unless it is being puffed.

Modifying cigarette design has the potential to change the chemical composition of cigarette toxin constituents [9] or alter an individual's smoking behavior and thus, nicotine and toxicant exposure [10-12]. Recently, O'Connor and colleagues [13] examined the behavioral and chemical effects of switching smokers to RIP versions of three popular American brand cigarettes (Newport, Camel, and Marlboro). Overall, no changes in smoking behavior were observed and, although measurable increases in exposure to phenanthrene were seen in smokers after switching to RIP cigarettes, generally no significant differences in biomarkers of exposure were identified [13]. At the same time, the effects of RIP regulation on exposure may be different in Canada given known differences in the blend of tobacco and the emissions profile relative to American cigarettes [14,15]. Cote and colleagues [16] reported an evaluation of the impact of the Canadian RIP regulation on smoking behavior and exposure using 'yield-in-use' assessments of spent cigarette filters. They report no significant differences in tar and nicotine exposures when comparing smokers' samples collected before and after the law [16]. However, the samples tested were independent groups, rather than a cohort. Studies utilizing cohorts of smokers may be more conducive to evaluating effects of policy changes, as demographic characteristics of the sample are consistent over time [17].

The goal of this study was to use a 'pre-post' evaluation design to measure the impact of the Canadian RIP law on smoking behavior and cigarette toxicity in Canadian smokers. Specifically, the study sought to take advantage of the natural experiment presented by the implementation of the Canadian RIP law to examine the stability of smoking topography over a ten month time period, pre and post RIP law. The study also examined variations in biomarkers of exposure to nicotine and polycyclic aromatic hydrocarbons (PAH's) studied by O'Connor et al. [13] before and after the RIP regulations were put into effect.

## Methods

### Participants

Participants were initially recruited from July through September 2005, prior to the implementation of Canada's RIP law. Eligibility criteria included being aged 18 to 55 years, smoking at least 100 cigarettes in their lifetime prior to the study, and having no intention to quit smoking in the next 6 months. Eligibility was further limited to those who smoked any of four leading Canadian brands (Players, DuMaurier, Number 7, and Peter Jackson). At the time of the study, these brands constituted over 50% of the Canadian market. Individuals with serious health conditions and females who were pregnant or planning to become pregnant were excluded from the study. Participants were required to provide written consent to participate.

### Materials and methods

Participants were recruited from the Kitchener/Waterloo region of Ontario, Canada for a ten month study examining behavioral and biomarker changes following the implementation of Canada's new RIP law. The study design is illustrated in Table 1. Participants were asked to visit the laboratory four times over a ten month period, twice pre-RIP law and twice post-RIP law. Pairs of visits at pre and post-RIP periods were scheduled 24-hours apart.

**Table 1:** Study timeline for pre-post evaluation of smoking behaviors and biomarker levels from smoking cigarettes

Condition		Pre-RIP		Switch		Post-RIP	
**Visit**	1	Field	2	RIP Law Change	3	Field	4
**Product**	N	N	N	R	R	R	R
**Breath CO**	X				X		
**Saliva Cotinine**	X				X		
**Urine PAH** **Metabolites**	X				X		
**Topography**		X	X			X	X

N = non-compliant cigarette; R = RIP-compliant cigarette

The timeline represents the treatments given to participants on each day of the study; Ontario, Canada, 2005-06.

At visit 1, participants completed a 15-minute baseline survey assessing demographics (age, race, and gender) and smoking behavior with a focus on consumption for the previous 24 hours. Questions were also asked regarding perceptions on RIP cigarettes including taste, fire incidents, and re-lighting of cigarettes on self-extinguishment. Following the survey, both saliva and urine samples were taken as well as pre-smoking breath samples for alveolar carbon monoxide (CO) examination. Biomonitoring was conducted prior to the field analysis to avoid disturbance by simultaneously monitoring smoking topography and potentially altering behavior. Participants then smoked one cigarette and provided a post-smoking breath sample. Finally, participants were instructed on the procedures for the 24-hour field collection period, including the use of the CReSSmicro (Plowshare/Borgwaldt-KC, Richmond, VA) portable topography recording device, as well as being provided with a diary and container to store cigarette butts.

During the following 24 hours, participants were asked to smoke at least 5 cigarettes of their normal brand *ad libitum *through the CReSSmicro. They recorded the times of each cigarette smoked in the diary and saved each cigarette butt in the container provided in visit 1.

Participants returned to the lab the next day for visit 2, where they first completed a survey on their experiences using the CReSSmicro. Next, participants smoked one cigarette using the CReSSmicro in the presence of a laboratory assistant. The laboratory assistant scheduled post-RIP visits and reminded participants of follow-up procedures which were to occur after approximately 10 months, following the implementation of the RIP laws. Contact and back-up contact information was gathered in order to confirm follow-up visits and participants were given $50CDN for their time and effort. Over the next ten months, laboratory assistants maintained contact with participants via mail and phone to maximize retention during the follow-up period.

The second half of the study followed the same design: two laboratory visits separated by 24-hours. At the conclusion of visit 4, participants were debriefed and provided with an additional $50CDN for completing the post-RIP half of the study. The study protocol received ethics approval from the University of Waterloo and complied with the laws of the country in which they were performed.

### Experimental: Smoking behavior, exposure biomarker, and smoking measures

Measures for smoking behavior, exposure biomarkers, and smoking are described in detail by O'Connor et al. [13]. Briefly, smoking topography was assessed using the CReSSMicro device (Borgwaldt-KC, Richmond VA). Exhaled CO was measured after a 15-second breathhold using a Micro+ device (Bedfont Scientific Ltd., Kent, UK). Saliva specimens were examined for cotinine concentration at an outside laboratory (Salimetrics LLC, University Park, PA). Urine specimens were measured for concentration of hydroxylated metabolites of naphthalene, fluorene, phenanthrene, and pyrene at the National Center for Environmental Health, Centers for Disease Control and Prevention, using gas chromatography isotope dilution high resolution mass spectrometry (GC-IDHRMS) [18]. All biospecimens were stored at -80°C until study completion and tested simultaneously. Participants also completed questionnaires detailing number of cigarettes smoked as well as perceptions and beliefs regarding cigarette fires.

### Calculations

Differences in demographics and other smoking related variables between participants were assessed using frequency and descriptive measures. Changes in smoking behavior and biomarker exposure were examined using generalized estimating equations with law status (pre-RIP, post-RIP) as a fixed within-subjects factor. The model controlled for age (as a continuous variable), race (white, other), gender (male, female), and brand (Players, du Maurier, Number 7, and Peter Jackson). Polycyclic aromatic hydrocarbon (PAH) biomarker concentrations were expressed on a creatinine adjusted concentration basis (ng/g creatinine). The concentration of PAH biomarkers formed from the same parent compound were summed, i.e., 1-hydroxynaphthalene (1-NAP) and 2-NAP were summed to form naphthalene (NAP); 2-hydroxyfluorine (2-FLUOR), 3-FLUOR, and 9-FLUOR formed fluorine (FLUOR); and 1-hydroxyphenanthrene (1-PHEN), 3-PHEN, and 4-PHEN formed phenanthrene (PHEN). 1-hydroxypyrene (PYR) was analyzed singly. Both PAH biomarkers and cotinine were natural logarithm transformed pre-analysis, hence only geometric means are reported. Statistical significance was accepted at *p*<0.05, two tailed, using SPSS Version 16 (SPSS Inc., Chicago, IL).

## Results

### Participants' characteristics

Demographic data for participants at the start of the study can be found in Table 2. Overall, the recruitment sample included a greater number of males to females (54.1% to 45.9%). 'Caucasian' participants predominated. The majority of participants smoked du Maurier cigarettes (44.3%). Individuals were on average 32 years old, smoking since approximately 15 years of age, an average of 18 cigarettes per day. All participants reported smoking for a minimum of four years. Though the number of those who completed the full study dropped by 31% (final n = 42), demographic characteristics of those who completed the study did not differ from the recruitment population on gender, brand, and age characteristics. Race information was only gathered post-RIP, so only those who completed the entire study were asked to reveal their race.

**Table 2:** Demographic characteristics of recruited participants (n = 61), Ontario, Canada, 2005-06

Variable	Level	Frequency	Percent
Gender	Male	33	54.1
	Female	28	45.9
Race	White	32	76.2
	Other	10	23.8
	Players	15	24.6
	du Maurier	27	44.3
Brand	Number 7	7	11.5
	Peter Jackson	9	14.5
	Other	3	4.9
		
	**Mean (SD)**		
		
Age	Years	32.6 (12.3)	
Age began smoking	Years	15.5 (2.9)	
Years smoking current brand	Years	8.1 (8.5)	
Cigarettes per day	N	18.0 (7.1)	
Time to first cigarette after waking	Minutes	39.9 (56.2)	

The table represents the demographics of the sample population at baseline by way of gender, race, brand, and age characteristics. Both raw frequency and percent out of the whole are depicted for categorical variables and mean values are represented for all continuous variables.

### Self-reported smoking behaviors

#### Self-reported cigarette self-extinguishment

At baseline, more than half (51.2%) of participants reported their cigarettes 'never' self-extinguished while smoking. However, after the RIP laws were put into effect, 24.4% reporting their cigarettes 'never' self-extinguished. These results were met by an increase in participants reporting that their cigarettes self-extinguished 'sometimes' only 14.6% pre-RIP regulations to 31.7% of the time post regulations. Overall, McNemar-Bowker's test revealed that difference in participants perceiving cigarettes to self extinguish after the RIP law (reports of Never, Sometimes, Always self-extinguishing pre vs. post law) was not statistically significant (p = 0.092).

#### Cigarette consumption - cigarettes per day (CPD)

Overall, there was no significant change in cigarette consumption before and after the RIP laws (M_CPD Pre-RIP _= 17.6, M_CPD Post-RIP _= 17.1). After the RIP laws were put into effect, although minimum CPD decreased by 2 cigarettes and maximum CPD increased by 2 cigarettes, the difference was not statistically significant (p = 0.724). After removing potential outliers, which were defined as any change > 10 cpd in either direction, we saw similar results (M_CPD Pre-RIP _= 13.7, M_CPD Post-RIP _= 14.9; p = 0.191).

### Smoking topography

Mean values pre and post law are shown in Table 3. Overall, there were no significant difference between pre and post law time periods on any topography variables. A significant effect on puff count was observed for race and brand, with all other individuals taking more puffs than white participants (B_Other _= 2.50, p = 0.023) and Peter Jackson smokers taking significantly fewer puffs than Players smokers (B_Peter Jackson _= -4.15, p = 0.002). A trend in race was also observed for average flow, and statistical significance was revealed for duration of puff; such that white participants smoked less intensively on the measures of velocity (B_Other _= 4.42, p = 0.052) and more intensively on duration of puff (B_Other _= -349.68, p = 0.006). Inter-puff interval and volume, on the other hand revealed significant differences in gender and brand such that females waited shorter amounts of time in between puffs compared to males (B_Females _= -3602.56, p = 0.026), but took in marginally less volume per puff (B_Females _= -8.97, p = 0.040). Peter Jackson, Number 7, and du Maurier smokers all waited longer amounts of time in between puffs compared to Players smokers (B_Peter Jackson _= 13291.73, p < 0.001; B_Number 7 _= 5629.60 p = 0.032; B_du Maurier _= 5495.17, p = 0.003). There were no differences between any covariates on the measure of total volume (p > 0.1). Age was not significant association with any topography variable. Adjusting for cotinine had no effect on any observed topography variable.

**Table 3:** Model-adjusted mean smoking topography characteristics (n = 42), Ontario, Canada, 2005-06

Topography Variable	Pre (95% CI)	Post (95% CI)	Change	B	*p*
Total Volume	834.9 (733.1, 936.7)	831.7 (745.8, 917.6)	0.30%	-3.17	0.95
Puff Count	14.1 (12.7, 15.5)	14.2 (12.6, 15.9)	1.20%	0.17	0.815
Per Puff Volume (mL)	59.6 (54.2, 64.9)	60.3 (54.9, 65.8)	1.30%	0.76	0.712
Puff Velocity (mL/sec)	36.8 (33.8, 39.8)	37.2 (34.9, 39.6)	1.20%	0.45	0.738
Duration of Puff (sec)	1.7 (1.6, 1.9)	1.7 (1.6, 1.9)	1.20%	20.66	0.719
Inter Puff Interval (sec)	19.2 (17.0, 21.4)	17.9(15.1, 20.6)	-7.20%	-1376.49	0.331

***Note: ***Model adjusted for age, gender, race/ethnicity, and brand

The above table represents the average values, percent change Pre and Post RIP compliance, B and statistical significance for all topography variables. The model is adjusted for age, gender, race, and brand covariates. All participants who did not complete the study were excluded for analysis.

### Exposure biomarkers

#### Carbon monoxide (CO)

A non-significant increase in exhaled CO was shown between the two time points. Mean values are shown in Table 4. There was a significant difference in brand such that participants who smoked Number 7 Reds showed higher CO levels than participants who smoked Players (B = 2.09, p = 0.027). There were no additional differences in gender, race, or brand. However, we did observe as individuals aged, their alveolar CO also increased (B_age _= 0.12; p < 0.001). As expected, total volume of smoke was positively associated with CO (B = 0.002, p < 0.05). Adjusting for cotinine had no effect on observed CO.

**Table 4:** Model-adjusted mean saliva and urinary biomarker levels pre- and post-law, Ontario, 2005-06

Biomarker	Pre (95% CI)	Post (95% CI)	Change	B	*p*
Breath CO (ppm)^1^	4.6 (3.7, 5.4)	4.8 (3.9, 5.7)	4.3%	0.26	0.580
Saliva Cotinine^2^ (ng/mL)	303.9 (242.0, 381.7)	351.1 (291.6, 422.6)	15.5%	0.14	0.126
Urine Naphthols^2^ (NAP)	20046.3 (16281.8, 24681.3)	22904.8 (19809.2, 26481.5)	14.3%	0.13	0.209
(ng/g creatinine) Urine					
Hydroxyfluorenes^2^ (FLUOR)	2616.3 (2121.1, 3226.7)	3196.1 (2651.3, 3852.6)	22.2%	0.20	***0.013***
(ng/g creatinine) Urine					
Hydroxyphenanthrenes^2^ (PHEN)	469.1 (391.4, 562.3)	546.6 (458.2, 652.0)	16.5%	0.15	0.061
(ng/g creatinine) Urine					
1-hydroxypyrene ^2^ (PYR) (ng/g creatinine)	162.4 (124.0, 212.7)	200.6 (155.6, 258.6)	23.5%	0.21	***0.018***

***Notes: ***Means adjusted for age, gender, race/ethnicity, brand, total volume, IPI, velocity

1. Arithmetic mean

2. Geometric mean

The table above shows the geometric average values, percent change Pre and Post RIP compliance, B and statistical significance for all biomarker variables. The model is adjusted for age, gender, race, brand, and topography covariates. All significant changes are noted by bold *p *values. All participants who did not complete the study were excluded for analysis.

#### Cotinine

We found no significant difference in cotinine after the RIP law was put into effect. Mean values are displayed in Table 4. Again, as expected, we did observe an association between total volume and cotinine (B = 0.001, p < 0.05). Since smokers were free to smoke *ad libitum *throughout the course of the study, we have no reason to believe that this change could be due to alterations in cigarette consumption directly before the study session. Additionally, we found no significant difference in cotinine for any other covariates the model controlled for.

#### Polycyclic aromatic hydrocarbon metabolites (PAH)

Results showed small but statistically significant increases in most PAH biomarker concentrations on a creatinine adjusted concentration basis after the implementation of the RIP law. Mean values are depicted in Table 4. Urinary metabolites of fluorene (FLUOR) and pyrene (PYR) significantly increased at follow-up (B_After Lip Laws _= 0.20, p = 0.013; B_After Lip Laws _= 0.21, p = 0.018), while the increase of phenanthrene (PHEN) metabolites was notable, but nonsignificant (B = 0.15, p = 0.061). Napthalene (NAP), FLUOR, and PHEN PAH biomarker metabolite values revealed a significant brand difference such that people who smoked Peter Jackson, Number 7, and du Maurier cigarettes exhibited lower PAH levels than Players (p < 0.05). PYR showed a significant brand difference in that Peter Jackson and Number 7 smokers exhibited lower PYR levels than Players smokers (p < 0.01). Additionally, there was a marginally significant difference by race for NAP metabolites. NAP values were lower in all other participants than white individuals (B_Other _= -0.40, p = 0.024). There were no differences in gender, age, or topography for any biomarker values (p > 0.1). Further tests on the individual NAP biomarkers revealed that, although 2-NAP increased marginally after the RIP laws (B = 0.18, p = 0.053), 1-NAP did not (B = 0.11, p = 0.378). Summed results were non-significant (B = 0.13, p = 0.209). Adjusting for cotinine had no effect on findings related to PAH biomarkers.

## Discussion

The goal of this study was to examine if switching smokers from conventional cigarettes to RIP - standard cigarettes would produce a change in smoking behavior and/or biomarker level. However, unlike an experimental switching design, this study allowed for the observation of smokers naturally adopting use of the modified products. Any substantial change in either behavior or biomarker value could have implications on the cigarette's health risk to the smoker. The results indicate that the RIP cigarette design change did not lead to alterations in either cigarette consumption or smoking topography. This disconfirms the hypothesis that smokers switching to RIP cigarettes would puff considerably more intensively or consume more cigarettes to compensate for the self-extinguishing feature of RIP compliant cigarettes.

That being said, urinary PAH biomarker values were consistently elevated after the cigarette design change (14.3-23.5% higher), adjusting for topography and demographics. Hydroxyfluorene (FLUOR) and 1-hydroxypyrene (PYR) increased significantly (22.2% and 23.5% increase respectively). Though the increase in hydroxyphenanthrene (PHEN) was statistically non-significant, there was observed a notable increase (16.5%, p = 0.061). This is a concern because of the association of these biomarkers with benzo[a]pyrene, a known human carcinogen. The magnitudes of change are higher than that observed by O'Connor et al. [13] in American smokers, though the mean biomarker values are in the same range. However, unlike the American study, the observed increases in Canada were consistent across all biomarkers tested. These inconsistencies between studies may reflect differences in the study designs (naturalistic versus experimental) and/or duration of observation (10 months versus 18 days). The broader toxicological implications of these results are unclear. At least one study has demonstrated that there is no difference in toxicology both in vitro (genotoxicity or cytotoxicity) and in vivo (dermal promotion studies with mice) between traditional and banded RIP cigarettes [19]. In addition, While Brzeznicki and colleagues determined the half-life of urinary 1-PYR to be 9.8 hours, half-life information on other PAH biomarkers is scarce [20,21]. Given the OH-PAH metabolites in this study are formed by similar biological pathways, we can suspect that they also share a similar half-life to PYR [21]. Thus, we do not believe that any change in metabolites is due to length of time between cigarette and time of sample. However, changes in exposure levels could reflect modifications to cigarette design that were unrelated to RIP laws. Tobacco companies have the opportunity to change the tobacco blend and other design characteristics of their cigarettes at any point. Further studies are needed with a larger number of subjects before any firm conclusions can be drawn.

Although this study allowed for observation using a clean pre-post study design in both laboratory and natural settings, there were several limitations. A large limitation included the lack of mainstream smoke emissions data and toxicity reports directly assessing differences based on design change. Though the scope of this study was biobehavioral, future research should examine the emissions of the cigarettes themselves and assess the extent to which these could contribute to altered PAH levels. This could provide further insight into the actual toxicity of the cigarettes pre and post RIP regulation. Second, since the final sample size was low (N = 42), it may not be representative of the total Canadian population of smokers. We adjusted any differences in demographics by controlling for age, gender, race, and brand of cigarette smoked. Third, our study spanned a 10 month time period, and while participants were still living in the Waterloo area, we cannot guarantee that their exposures to other sources of PAH (e.g., diet, local environment) were consistent at both time points. Finally, we only examined leading Canadian cigarette brands, including Peter Jackson, Number 7, du Maurier, and Players. Since this study, the market for contraband cigarettes smuggled into Canada has grown considerably, particularly in Ontario. Thus, since the gain in popularity, it may be worthwhile to take contraband cigarettes into account. While Health Canada testing has showed these cigarettes to have similar emissions profiles as leading brands, testing for RIP compliance has not been reported [22].

## Conclusions

Overall, we found little change in both behavior and exhaled CO levels when switching smokers from traditional to RIP cigarettes. We did observe significant increases in PAH biomarker levels (22.2% in FLUOR and 23.5% in 1-PYR). Toxicological implications of these changes are currently unknown. Although RIP cigarettes are primarily designed to reduce fire risk, considerations regarding heath implications of cigarette design change should also be taken into account.

## List of abbreviations

CO: carbon monoxide; CPD: cigarettes per day; FLUOR: sum of concentrations of 2-, 3-, & 9-hydroxy fluorine expressed as ng/g creatinine; NAP: sum of concentrations of 1-,& 2-hydroxy naphthalene expressed as ng/g creatinine; PAH: polycyclic aromatic hydrocarbons; PHEN: sum of concentrations of 1-, 3-, & 4-hydroxy phenanthrene expressed as ng/g creatinine; PYR: 1-hydroxy pyrene expressed as ng/g creatinine; RIP: reduced ignition propensity.

## Competing interests

The authors declare that they have no competing interests.

## Authors' contributions

RJO, DH, and AS designed the study. ZL and LR led the laboratory analyses. KJ led data analysis and wrote the first draft. All authors contributed to manuscript drafting and approved the final version.
